# The Association between Postpartum Pelvic Girdle Pain and Pelvic Floor Muscle Function, Diastasis Recti and Psychological Factors—A Matched Case-Control Study

**DOI:** 10.3390/ijerph19106236

**Published:** 2022-05-20

**Authors:** Małgorzata Starzec-Proserpio, Montserrat Rejano-Campo, Agata Szymańska, Jacek Szymański, Barbara Baranowska

**Affiliations:** 1 Department of Midwifery, Centre of Postgraduate Medical Education, 01-813 Warsaw, Poland; barbara.baranowska@cmkp.edu.pl; 2 Montse Rejano Physiotherapist, Paseo Marítimo los Charcones 9, 35414 El Puertillo, Spain; montserejano@gmail.com; 3 Department of Rehabilitation, Faculty of Medical Sciences, Medical University of Warsaw, 02-109 Warsaw, Poland; agata.szymanska@wum.edu.pl; 4 First Department of Obstetrics and Gynecology, Centre of Postgraduate Medical Education, 01-004 Warsaw, Poland; jacek.szymanski@cmkp.edu.pl

**Keywords:** pelvic girdle pain, pelvic floor, pelvic floor disorders, rectus abdominis, biopsychosocial model

## Abstract

There is uncertainty regarding the association between abdominal morphology, pelvic floor function, and psychological factors in women with postpartum pelvic girdle pain (PGP). The aim of this case-control study was to evaluate the differences between women with and without persistent PGP regarding pelvic floor function, diastasis recti, and psychological factors 6–24 weeks postpartum. Pelvic floor manometry, palpation examination of abdominal muscles, the International Consultation on Incontinence Questionnaire Short Form, The Depression, Anxiety and Stress Scale—21, and the Pain Catastrophizing Scale were used. The PGP group presented with lower vaginal resting pressure (*p* < 0.001), more tenderness (*p* = 0.018) and impaired voluntary activation of pelvic floor muscles (*p* ≤ 0.001). Women with pain also had more distortion on the level of the anterior abdominal wall (*p* = 0.001) and more severe diastasis recti (*p* = 0.046) when compared to pain-free controls. Lower vaginal resting pressure was the strongest factor explaining PGP (OR 0.702, 95%CI 0.502–0.981). There were no differences in terms of the pelvic floor strength, endurance, severity of urinary incontinence and reported distress between the groups. Women with PGP 6–24 weeks postpartum differ in pelvic floor and abdominal muscle function from the pain-free controls. Vaginal resting pressure may be an important factor in pelvic girdle pain shortly postpartum. Further studies are needed to see a trend in changes over time.

## 1. Introduction

Pelvic girdle pain (PGP) is defined as pain located between the posterior iliac crest and the gluteal folds, particularly in the area of the sacroiliac joints and/or the pubic symphysis. It may also radiate to the posterior thigh, and pain in the inguinal area may appear. Patients with PGP have difficulties in maintaining prolonged standing or sitting positions, as well as transitioning from sit to stand or rolling in bed [1]. Symptoms of PGP are commonly reported to healthcare providers by pregnant or postpartum women. The pain often subsides after delivery, but some women continue to have persistent symptoms postpartum. Women experiencing PGP report that the pain had unexpected impact on their daily life. [2] A combination of hormonal and biomechanical aspects, inadequate motor control, and stress on ligament structures are the most common hypotheses behind the development of PGP [1,3]. More recently, central pain mechanisms have been considered and implicated [4].

There is a body of evidence demonstrating that PGP is a distinct condition from low back pain (LBP) and pelvic pain and should be studied separately [1]. To date, there are few studies that focus on PGP specifically. Many of them include heterogeneous groups, analyzing women with PGP grouped together with women having other lumbopelvic pain conditions like low back pain or abdominal/pelvic pain, not necessarily having PGP at the same time [5,6,7,8,9,10].

The pelvic floor muscles (PFM) and abdominal muscles play an essential role in the stabilization and motor control of the pelvis. Additionally, greater separation of rectus abdominis muscle bellies—diastasis rectus abdominis (DRA)—has been suggested to constitute a non-optimal myofascial system that fails to achieve optimal strategies for transferring loads through the abdominal canister, including the bony structures, pelvic floor muscles, diaphragm, and abdominal muscles [11]. Abdominal muscle force might be affected in women with DRA [12]. Therefore, the current physiotherapy practice in the management of PGP includes pelvic floor and abdominal muscles exercises and relaxation [13,14]. However, the rationale to support this kind of intervention is still unclear since the association between PGP and disturbances in the pelvic floor and abdominal muscle separation seems to be conflicting in the literature.

In the same way that PFM dysfunction and abdominal muscles separation can have an impact on the ability to stabilize the trunk, so do the morphological changes of the connective tissue in the abdominal region. The excessive elongation of the linea alba has been related to a loss of abdominal muscles strength [15]. For this reason, we believe it is necessary to deepen the research on the muscular response in women with PGP. Furthermore, only few studies carried out so far included a population of women in early postpartum stage. The muscular response may be different in women after delivery compared to women further postpartum.

Until now, the management of PGP has mostly focused on the treatment of physical impairments, with a relative lack of broader psychosocial aspects [13]. However, from a patient-centered perspective, psychosocial factors are essential in the management of musculoskeletal disorders, especially when treating conditions involving persistent pain. A comprehensive approach should consider both biomechanical factors and social and psychological aspects. In fact, in the latest recommendations for the management of musculoskeletal pain, Lin et al. [16] emphasize the necessity of considering biopsychosocial aspects in these patients. Other authors also support the need to evaluate patients with PGP from a biopsychosocial perspective [4,17].

Moreover, it is known that psychological factors may modify pain perception [18,19]. In addition, muscle function may be altered in the presence of psychological factors like pain catastrophizing [20] or depression [21].

To date, no study has combined the assessment of abdominal morphology, pelvic floor muscle function, and psychosocial factors in women with PGP within the first 6 months postpartum. Therefore, the aim of this study was to assess the differences in women with and without clinically diagnosed PGP 6–24 weeks postpartum from a biopsychosocial perspective by investigating the associations between PGP and pelvic floor function, diastasis recti, and psychological factors.

## 2. Materials and Methods

### 2.1. Study Design

The study was designed as a one-to-one matched case-control study. Each woman with PGP was matched to a woman without PGP by age (±5 years), time after delivery (±4 weeks), type of delivery, and number of previous deliveries. Ethics approval was received from the Bioethics Committee of the Medical University of Warsaw (KB/136/2017) and was registered at https://clinicaltrials.gov/ under number NCT04757077. The data was collected between February 2021 and May 2021. All the participants provided written informed consent prior to commencing any of the study procedures. The study was supported by the Department of Midwifery at the Centre of Postgraduate Medical Education under a Research Program for 2021. The Strengthening the Reporting of Observational studies in Epidemiology (STROBE) checklist was followed to ensure proper reporting of this case-control study (Appendix A).

### 2.2. Participants

The cases that were included were women between 18 and 45 years old, 6–24 weeks postpartum, and reporting pain in the pelvic girdle region, confirmed by indicating their pain on a body chart. They were recruited from our previous study on postpartum PGP prevalence and from among patients from gynecological offices and pelvic health physiotherapy offices by means of leaflets, verbal information, and social media posts. For PGP classification, existing guidelines and previous reports were used [1,22]. The examination consisted of functional tests dedicated to pelvic girdle assessment. At least two tests had to be positive to allocate the participant in the PGP group: the posterior pelvic pain provocation (P4) test, the distraction test, the compression test, palpation of the pubic symphysis, the modified Trendelenburg test, the FABER test, and palpation of the long dorsal ligament. When assessing the pain provocation tests, it was recorded if a familiar pain was provoked. Patients with confirmed PGP assessed the pain intensity on a numerical rating scale (NRS) from 0 to 10, where 0 meant no pain and 10 meant the worst pain imaginable. Functional limitations were assessed with the Polish version of the Pelvic Girdle Questionnaire (PGQ) [23]. Only women with PGP symptom onset during a pregnancy or within three weeks postpartum were included.

The control group consisted of women without complaints of pain in the pelvic girdle region. They were recruited from among patients from gynecological offices and pelvic health physiotherapy offices by means of leaflets, verbal information and social media posts. Apart from the presence of pain, all the remaining inclusion criteria were the same as for the PGP group. The exclusion criteria for both groups were other diseases that may mimic PGP (rheumatoid arthritis, ankylosing spondylitis, Scheuermann disease, Ehlers–Danlos syndrome, spinal surgeries, and spondylolisthesis, visible skeletal deformities and leg length discrepancies, pain caused by fractures, direct trauma, or neoplastic processes), and a positive neurological examination (nerve root compression and sensory deficits). All of the participants spoke and understood Polish.

### 2.3. Variables/Outcome Measures

#### 2.3.1. Pelvic Floor Measurements

The pelvic floor muscle (PFM) assessment was performed using vaginal manometry (Peritron™ 9300 V, Laborie, Mississauga, ON, Canada). It consisted of an air-filled silicone rubber sensor connected to a measuring unit via a plastic tube. The unit measured pressure in cmH_2_O. The sensor was covered with a sterile latex sleeve for each patient, and a small amount of water-soluble lubricant was applied. During the pelvic floor measurement, three values were recorded: PFM tone (vaginal resting pressure), strength (vaginal pressure during maximal voluntary contraction (MVC)), and endurance (area under the curve during a 10 s contraction).

Before the examination, the women were asked to empty their bladder to ensure their comfort during the assessment and to avoid any potential influence of bladder fullness on PFM activity. They were then asked to lie down, with their knees bent. Digital palpation was always the first examination carried out to confirm the woman’s ability to contract the PFM. The following was recorded: relaxation (correct, partial or absent) and ability to correctly activate the pelvic floor muscles. Isolated pelvic floor contraction without breath holding was considered as correct. In case of incorrect pelvic floor activation, a short instruction for correction has been provided to secure valid manometric measurements.

After ensuring proper PFM contraction, the vaginal sensor was inserted into the vaginal canal to the full extent of the compressible portion of the device until it was above the level of the hymenal ring. The baseline vaginal resting pressure was then recorded. The value was read after about 30 s. This was done to avoid measurement bias due to the changed muscle length and possible reflexive increase of resting pressure upon probe insertion. The reading after insertion depends on several factors and will be individual for every patient. To compensate for this variability, before measuring the MVC, the sensor was inflated to a preset value of 100 cmH_2_O, as suggested by the manufacturer. Then, the device was reset to zero and the vaginal pressure during MVC was measured. The women were asked to contract their PFM in and up as strong as possible for 3 s, 3 times. We used an interval of 10 s between contractions. The device was reset to zero after each contraction. The mean of the three trials was recorded. Following that, after a 30 s break, the ability to sustain near maximal or maximal contraction was assessed and quantified as the area under the curve for ten seconds, measured on one attempt. This value was previously used as a quantification of muscular endurance in another study [24]. Valid measurements were ensured by the simultaneous observation of the inward movement of the perineum. Any contractions for which a retroversion of the hip or a Valsalva maneuver was noticed were discounted.

The vaginal squeeze pressure during PFM contraction has shown excellent test–retest (ICC = 0.88–0.96) [25] and intrarater reliability (r = 0.88) [26] for the assessment of muscle strength and endurance. In support of the validity of manometry, maximal pressure measurement has been found to be correlated with vaginal palpation—the modified Oxford scale (r = 0.646) [27] and transabdominal ultrasound (r = 0.72) [28].

#### 2.3.2. Urinary Incontinence

The International Consultation on Incontinence Questionnaire Short Form (ICIQ-UI SF), polish version, was used for the assessment of urinary incontinence presence and severity. The tool is considered by the International Consultation on Incontinence as a gold standard outcome measure in clinical practice and research [29].

#### 2.3.3. Diastasis Recti Assessment

The diastasis recti was measured by palpation. The assessments were performed 2 cm over the umbilicus [30]. The participants were positioned supine, and the desired measurement location was marked with a water-soluble pen. The palpation examination procedure was taken from previous report [30]. The women were asked to perform an abdominal curl-up by raising their head and upper torso until their shoulder blades left the examination bed. The physiotherapist placed her fingers across the linea alba at the level of the marked point so that the width of her fingers would fill the gap between the edges of the abdominal rectus muscles. According to other studies implementing this method [6,31], diastasis recti abdominis (DRA) was considered when the inter-recti distance (IRD) was ≥ 2 fingerbreadths. The participants in this study were divided into four categories depending on the largest palpation measurement (number of fingers) in one of three locations: no DRA (IRD < 2 fingerbreadths), mild DRA (IRD 2; < 3), moderate DRA (IRD 3; < 4), and severe DRA (IRD > 4) [31]. Mota et al., showed good intrarater reliability (K_w_ > 0.70) in terms of the palpation measurements of the IRD [30], and Benjamin et al. showed moderate to very good correlation of IRD palpation with ultrasound (r = 0.75–0.98) [32]. Additionally, the distortion/bulging in the projection of the linea alba were assessed. This was defined as the “abdominal midline stability” (stable/distorted). The bulging of the abdominal surface in the projection of the linea alba was considered as “distorted.”

#### 2.3.4. Psychological Factors—Distress Questionnaires

The Depression, Anxiety and Stress Scale-21 Items (DASS-21) was used to assess the self-reported emotional states of depression, anxiety and stress. A recent Delphi Study indicated this tool as a one of the preferred instruments to use in people at risk of developing or maintaining persistent musculoskeletal pain [33]. The Pain Catastrophizing Scale (PCS) was used to assess the mental processing associated with experiencing pain. Higher scores indicate a higher level of catastrophizing. This questionnaire is one of the patient-reported outcomes indicated for use with patients with PGP [4].

All the examinations were performed by the same registered pelvic health physiotherapist with clinical experience in women’s health. She has completed advanced training in urogynecology and has been certified by the Polish Urogynecological Society to digitally examine pelvic floor muscles. She was also involved in data analysis.

### 2.4. Statistical Analysis

We based the sample size calculation on the vaginal resting pressure value. We aimed to detect the difference between group means of at least equal to the common standard deviation. With 95% power, an allocation ratio of 1, and for a 5% significance level, at least 54 participants should be included (27 in each group (G*Power 3.1)). Considering the possibility of missing data, we included 56 participants.

The means and standard deviation (SD) were calculated for continuous variables. Categorical data are presented as counts and percentages. Continuous variables were compared across subjects with and without pain using the Student’s *t*-test or the Mann–Whitney U test, depending on whether data distribution was normal. Categorical variables were compared through the chi-square test. A special Cox regression model was used to fit a conditional logistic regression procedure for one-to-one matched case-control studies. Multivariable logistic models were used to identify associations with more than one factor included in the models. The selection of factors to include in the multivariable models was informed by the univariable results. The best subset of explanatory variables was selected manually by excluding the variables with the smallest contribution to the model. The results are given as odds ratios (OR) with 95% confidence intervals (CI). Due to the design of the study, all the analyses were adjusted for age, parity, type of delivery, and time after delivery. Missing data were not included in the analysis. The outcomes were considered statistically significant when *p* < 0.05. Statistical analyses were performed using PQStat software version 1.8.2.144.

## 3. Results

Twenty-eight women with clinically confirmed PGP were successfully matched to 28 controls according to age, parity, type of delivery, and time after delivery. Both groups did not differ in terms of age, parity, body mass index (BMI), weight gain during pregnancy, and completion of higher education. However, PGP during pregnancy was statistically more often reported in the PGP group when compared to the controls. The characteristics of the study groups are presented in Table 1. As shown in Table 2, women with PGP were afflicted by pain and functional limitations.

Women with PGP had statistically significantly more PFM tenderness, more difficulties in performing a correct PFM voluntary contraction, presented more often with a higher degree of diastasis recti and more distortion in the abdominal midline (in the projection of the linea alba), and had lower vaginal resting pressure when compared to the controls. There were no statistically significant differences between the groups regarding PFM relaxation, vaginal squeeze pressure and endurance (expressed as an area under the curve during the manometric measurement). Additionally, both groups did not differ in terms of urinary incontinence or the results of the distress questionnaires (Table 3). The results of multivariable regression analysis are presented in Table 4. The value of the manometric measurement of the vaginal resting pressure was the strongest variable predicting PGP. There were no missing data.

## 4. Discussion

The presented one-to-one matched case-control study showed that women with PGP 6–24 weeks postpartum presented with lower vaginal resting pressure, impaired voluntary PFM activation, and more PFM tenderness. They also had more distortion on the level of the anterior abdominal wall and more severe DRA when compared to women without pain. However, women with PGP did not differ in terms of the PFM strength, endurance, and severity of urinary incontinence from the healthy controls. Additionally, both groups had similar levels of self-reported stress, anxiety, depression, and pain catastrophizing.

### 4.1. PFM Tone

The main difference in our study when compared to previous reports refers to the PFM tone. In our study, women with PGP presented with lower vaginal resting pressure, while previous studies reported a tendency for a higher PFM tone. Stuge et al. [34] reported a higher PFM muscle tone described as a smaller levator hiatus area when measured by 3D ultrasound. Our study group was relatively recently postpartum, while Stuge et al. included women who were at least six months after delivery (with a mean duration of symptoms of 3.4 years). The early postpartum period is a very specific context during which many hormonal changes occur. It is known that endogenous hormonal factors are associated with muscle tone changes. For example, lower levels of estrogen are related to lower muscle tone and muscle mass [35,36]. The differences in the postpartum state (early versus late postpartum) could explain the disparities between our results and those of Stuge et al. [34].

Another hypothesis that may explain such differences may be associated with the measurement tools. Stuge et al. [34] found increased muscle tone when assessed by ultrasound, but the differences in the manometric measurements were not significant. Braekken et al. [37] found that muscle function (resting tone and strength) explained only 26% of the variance in the levator hiatus area when measured by ultrasound in women with pelvic organ prolapse. This shows that 3D ultrasound may evaluate different components of PFM tone, and that manometry values may be affected by vaginal tissue distensibility and/or paravaginal connective tissue. Thus, a woman may present with a high tone and elongated fascial tissues at the same time.

Pool-Goudzwaard et al. [7] also reported a higher muscle tone measured by palpation in patients with pregnancy-related PGP and low back pain. However, they used different assessment criteria. They considered the participants as having an “increased tone” when they were not able to relax the PFM immediately after a contraction or when an increase of the muscle tone during/after flick contractions occurred. They did not include any validated quantification of the muscle tone but a subjective observational assessment. This assessment method is difficult to compare with other studies assessing PFM tone at rest. In this study, the differences between groups in muscle tone were not statistically significant when measured by electromyography.

Our results have shown that women with PGP early postpartum (6–24 weeks) may present with lower vaginal resting pressure, lower ability to activate PFM, and PFM tenderness. Compared to results reported by other studies, we hypothesized that women with postpartum PGP may initially present with lower muscle tone and lack of movement/disuse of PFM (due to pain, tenderness, and impaired activation). Subsequently, this could lead to adaptive changes in their passive structures and result in viscoelastic stiffening [38]. This could eventually present as an increased stiffness of PFM that could then be measured as increased muscle tone further postpartum.

### 4.2. PFM Tenderness

In terms of muscle tenderness, the results of our study confirm previous reports where participants with PGP presented more often with pain/tenderness at the level of the PFM assessed by palpation [39,40] or the Urinary Distress Inventory (UDI) questionnaire [7]. Sensitivity to light touch/muscle tenderness has been previously linked to central sensitization [41]. Current advances in pain science suggest that pregnancy-related PGP may represent the sensitization of the structures of the pelvis, indicating a different approach to pain management and physiotherapy [4].

### 4.3. PFM Strength and Endurance

When it comes to PFM strength, our results were consistent with previous reports. The strength of PFM has been studied by many research groups [7,34,39,42,43,44]. Different tools were used to measure this variable, namely, manometry [34,42,44] and intravaginal palpation [7,39,43]. No differences regarding muscle strength have been found between groups.

Regarding endurance, our results are similar to those described by Stuge et al. [34], who measured PFM endurance through manometry and did not report any differences between groups. However, the study carried out by Pool-Goudzwaard et al. [7] found statistically significant shorter contractions in women with LBP/PGP than in subjects in the control group. This team measured the endurance capacity of the contraction at 50% of the maximal voluntary contraction using surface electromyography. Since both measurement methods (manometry and electromyography) assess different parameters of muscle endurance (bioelectrical activity vs. vaginal pressure), it may be possible that women with PGP have a lower capacity to keep the PFM contraction, but this change may be not associated with changes in vaginal pressure.

### 4.4. Ability to Voluntary Activate PFM

The PGP group in our study presented with more difficulties in the correct voluntary activation of the PFM when compared to the controls. Fitzgerald et al. [43], assessing pregnant women with PGP by palpation, did not find such differences. However, they were assessing the ability to voluntarily contract the pelvic floor as either normal (present) or abnormal (absent, impaired, unable). In our assessment, only an isolated pelvic floor contraction without breath holding, which requires more advanced coordination, was considered correct and could explain the differences in the results obtained.

### 4.5. Psychological Factors

In our study group, women with PGP 6–24 weeks postpartum did not differ in terms of psychological factors from the healthy controls. Similarly to our results, Rexelius et al. [45] reported depressive symptoms to be similar between women with postpartum PGP and healthy controls 3–12 months postpartum. The authors hypothesized that a longer period of PGP is required to affect psychological wellbeing than other types of pain like, for instance, low back pain. However, these results seem to be contrary to previous studies on PGP. It has been reported that women with PGP present with more depression, anxiety, and pain catastrophizing than healthy controls during pregnancy and postpartum [22,46,47], and a recent systematic review has shown that depression during pregnancy may be a risk factor for ongoing pain postpartum [9].

One of the reasons explaining these differences may be the time after delivery in our study group. Although our group consisted of women between 6 and 24 weeks postpartum, the mean time after delivery was 11 weeks. The first weeks postpartum may be challenging and distressing for many women despite pain; therefore, the differences in psychological factors may not be so evident. Previous studies reporting a connection between psychological factors and PGP were measuring distress during pregnancy [46] or up to a period of 11 years after it [22]. Although Gutke et al. [47] found a higher prevalence of depressive symptoms among women with lumbopelvic pain three months after delivery, they reported a stronger association with depressive symptoms for lumbar pain than for PGP. Another reason explaining the differences is the measurement tool applied. We used DASS-21 for the assessment of depressive and anxiety symptoms, while other studies reporting an association between these factors and PGP used the State-Trait Anxiety Inventory [46], the Beck Depression Inventory [46], and the Edinburgh Postnatal Depression Scale [47]—tools that may be more specific than screening with DASS-21. What is more, PGP is not commonly recognized in Poland, and the term “pelvic girdle pain” is not widely used within healthcare services. The lower social awareness about this condition could lead to lower reported distress. The possible role of ethnicity was noticed in another PGP study [48], indicating that a more detailed investigation encompassing the cultural and ethnic influences associated with PGP is needed.

### 4.6. Urinary Incontinence

In our study group, there were no differences in the reporting of urinary incontinence symptoms between women with and without PGP 6–24 weeks postpartum. This is contrary to a recently published systematic review by Bertuit et al. [49] evaluating PGP, lumbopelvic pain, and pregnancy-related low back pain. It included four studies and all of them reported a significant association between urinary incontinence and pain. However, the inclusion criteria and the study groups of the mentioned studies differed from ours. In the study of Yi et al. [50], urinary incontinence was associated with a higher score on the Pelvic Girdle Questionnaire; however, the study group consisted of triathletes, in whom pelvic floor dysfunctions are highly prevalent. Fitzgerald et al. [43] and Mens et al. [51] studied pregnant women, which could lead to differences in the obtained results. Pool-Goudzwaard et al. [7] also studied postpartum women, reporting a higher prevalence of urinary incontinence in patients with pregnancy-related low back pain. However, although they included women who were at least 12 weeks postpartum, their mean age was higher than that of our group (35–42.6 years), suggesting that a much longer time could have passed from their last delivery. The longer pain duration may have influenced the obtained result. Additionally, Mens et al. [51] and Pool-Goudzwaard et al. [7] studied more heterogeneous groups, including women with PGP and women with low back pain too. It is possible that the prevalence of urinary incontinence is different in these subgroups of patients.

### 4.7. Diastasis Recti

In this study, women with postpartum PGP 6–24 weeks postpartum presented with a wider inter-recti distance and more distortion on the level of the linea alba than women without pain. This is partially in line with our previous matched case-control study among women with and without PGP 24–72 h postpartum [52]. In this report, the inter-recti distance and the bulging on the level of the linea alba were not different between women with and without PGP. As the separation of the rectus abdominis muscle bellies is highly prevalent in the early postpartum period [53], these differences could not have been visible. However, in multivariable analysis, women with a wider inter-recti distance measured by ultrasound during the curl-up task were having higher odds of experiencing postpartum PGP. Our reports seem to be contrary to the recently published systematic review by Fuentes Aparicio et al. [54] which, based on the included studies, did not find any correlation between the diastasis recti and the severity or the presence of lumbopelvic pain. It has to be noted that previous studies were investigating PGP grouped with other pain conditions such as abdominal pain [5], as a lumbopelvic pain entity [8], or as pelvic pain [5,6]. Our study included only women with clinically confirmed PGP, which makes it different from those previously reported. It is, therefore, possible that there are some associations between the compromised anterior abdominal muscle wall and the presence of PGP that are not evident in other lumbopelvic pain conditions. Additionally, in this study, the groups were very similar as we matched them according to age and parity—the main risk factors of diastasis recti [55]. Gluppe et al. [12] also matched their participants by age and parity. In their recently published report, they analyzed the problem from a different perspective, including women with DRA (n = 36) and without (n = 36). They did not find any association with PGP. However, over 90% of the study group were over six months postpartum. Considering that PGP persists until 18 months postpartum in less than 10% women [56], the association between DRA and PGP could not have been visible.

Although sensible from a theoretical point of view, there is currently no evidence supporting the statement that compromised PFM function and DRA impacts the stability of the pelvic girdle leading to PGP. However, it is possible that these conditions coexist, being related by certain common factors. In these circumstances, it is important to remember that pain is complex and multifactorial, and that there are a variety of biopsychosocial factors leading to its occurrence and persistence. This could explain the dissimilarity of results obtained in different studies.

### 4.8. Strengths of the Study

The strength of this study is the robust matched case-control design focusing on women with clinically diagnosed PGP. Although our study group could have other co-existing musculoskeletal pain conditions, all of them were classified with PGP using a clinical examination and the recommended guidelines. This makes our study different from the previously published reports with more heterogeneous study groups in which women with other lumbopelvic pain conditions were also included, therefore, not necessarily having PGP. All the assessments were provided by the same experienced physiotherapist and an objective, manometric assessment of the pelvic floor was carried out, which minimized the bias related to the measurements.

### 4.9. Limitations

The main limitation of this study relates to the palpation method used in abdominal wall assessment, while the current gold standard is an ultrasound assessment of diastasis recti [30]. However, palpation examination of diastasis is widely used in clinical practice [57], as well as in other research studies [6,31,32,58,59,60]. We used a non-validated visual assessment of the abdominal midline distortion, which does not allow the specific structure that is bulging to be determined. However, the used form of measurement aimed to inform future studies about the eventual need for further investigation. Additionally, the observation of diastasis bulging has already been used in other research [61]. Another limitation is the possible intra-examiner bias. The evaluating physiotherapist could not be blinded to patients’ diagnosis. We believe we have limited this issue to some extent by performing an objective manometric assessment of the pelvic floor and including patient reported outcomes (questionnaires). Finally, the majority of the included participants had a vaginal delivery which can impede the results from PFM examination and generalizability of the results. We believe to limit this bias to some extent by performing a matched analysis where women with PGP and post-cesarean section were matched to pain-free controls who were also post-cesarean section.

### 4.10. Implications

While no causal interferences can be made, our results may inform future studies about the possible associations and future directions of physiotherapy care while treating women with postpartum PGP. Given the potential association between changes in pelvic floor muscle function in women with PGP, in addition to the high prevalence of pelvic floor dysfunction in the early postpartum period, it makes sense for physiotherapists to individually assess PFM in women with PGP. This goes along with the proposed clinical practice guidelines for postpartum PGP where PFM assessment (including timing of contraction, strength, and resting tone), as well as DRA evaluation, have been recommended [62]. Future studies should focus on the assessment of multimodal physiotherapy programs, where the feasibility of the biopsychosocial approach implementing PFM and DRA therapy, together with psychosocial factors, will be evaluated.

## 5. Conclusions

This one-to-one matched case-control study among women with postpartum PGP (6–24 weeks after delivery) shows that women with pain present with lower vaginal resting pressure, impaired voluntary PFM activation, and an increase in PFM tenderness when compared with women without PGP. An increase of the inter-recti distance and a more common distortion (bulging) on the level of the anterior abdominal wall have also been found in women with PGP when compared to pain-free controls. All those differences reached statistical significance. However, women with PGP 6–24 postpartum did not differ statistically in terms of the PFM strength, endurance, and the severity of urinary incontinence from healthy controls. Similar levels of self-reported stress, anxiety, depression, and pain catastrophizing have been found in both groups. Further studies investigating the abdominal wall, pelvic floor function, and the psychosocial factors in specifically selected women with clinically confirmed postpartum PGP are needed to see a trend in changes over time.

## Figures and Tables

**Table 1:** Differences between cases with pelvic girdle pain (n = 28) and controls (n = 28).

			Cases	Controls	*p*-Value
Age in years, mean (SD) ^1^			31.61 (4.25)	31.04 (3.00)	*p* = 0.564
Parity, mean (SD)			1.43 (0.63)	1.43 (0.63)	*p* = 1.000
Mode of last delivery		vaginal, n (%)	22 (78.57%)	23 (82.14%)	*p* = 0.936
		vacuum extractor, n (%)	1 (3.57%)	1 (3.57%)	*p* = 0.936
		cesarean, n (%)	5 (17.86%)	4 (14.29%)	*p* = 0.936
Time after delivery in weeks, mean (SD)			11.00 (4.08)	10.96 (4.32)	*p* = 0.974
BMI, mean (SD)			23.30 (3.92)	22.71 (5.23)	*p* = 0.954
Weight gain during pregnancy, mean (SD)			14.14 (5.36)	11.95 (5.74)	*p* = 0.173
Education level	vocational and secondary education, n (%)		2 (7.14%)	1 (3.57%)	*p* = 0.553
	university education, n (%)		26 (92.86%)	27 (96.43%)	
Pelvic girdle pain during pregnancy (self-reported), n (%)			24 (85.71%)	14 (50%)	***p* = 0.004**

^1^ Normally distributed data—tested with the Student’s *t*-test. All the other continuous variables were not normally distributed and were tested with the Mann–Whitney U test.

**Table 2:** Characteristics of cases with pelvic girdle pain (n = 28).

		Mean (SD)	Number (Percentages)
Pelvic Girdle Questionnaire (0–100%)		34.77 (16.56)	
Numerical Rating Scale (0–10)		5.07 (1.74)	
Type of pelvic girdle pain	Posterior pelvic pain		6 (21.43%)
	Unilateral posterior pain		2 (7.14%)
	Symphyseal pain		8 (28.57%)
	Pelvic Girdle Syndrome (all 3 pelvic joints)		10 (35.71%)
	Unilateral posterior pain + symphyseal pain		2 (7.14%)

**Table 3:** Differences between cases with pelvic girdle pain (n = 28) and the controls (n = 28) with respect to the measured variables.

		Cases	Controls	*p*-Value
Tenderness of pelvic floor muscles (yes), n (%)		12 (42.86%)	4 (14.29%)	***p* = 0.018**
Correct activation of pelvic floor muscles (yes), n (%)		6 (21.43%)	19 (67.86%)	***p* < 0.001**
Relaxation after pelvic floor contraction (correct), n (%)		21 (75%)	24 (85.71%)	*p* = 0.313
Vaginal resting pressure (cmH_2_0), mean (SD) ^1^		29.78 (8.74)	41.23 (10.58)	***p*** **< 0.001**
Strength: Vaginal squeeze pressure (cmH_2_0), mean (SD)		17.50 (8.41)	21.78 (12.94)	*p* = 0.301
>Endurance: Area under the curve, mean (SD)		2402.96 (3438.10)	1945.60 (1697.86)	*p* = 0.833
Diastasis recti severity, n (%)	none, IRD < 2	15 (53.57%)	23 (82.14%)	***p* = 0.046**
	mild, IRD 2; <3	7 (25%)	5 (17.86%)	
	moderate, IRD 3; <4	2 (7.14%)	0	
	severe, IRD > 4	4 (14.29%)	0	
Abdominal midline stability, n (%)	Stable	19 (67.86%)	28 (100%)	***p* = 0.001**
	Distorted	9 (32.14%)	0	
International Consultation on Incontinence Questionnaire Short Form (0–21), mean (SD)		3.54 (5.46)	1.46 (2.47)	*p* = 0.295
Pain Catastrophizing Scale (0–52), mean (SD)		15.11 (9.78)	13.50 (7.38)	*p* = 0.718
Stress subscale of DASS-21 (0–42), mean (SD)		13.93 (8.35)	13.21 (8.90)	*p* = 0.616
Anxiety subscale of DASS-21 (0–42), mean (SD)		5.71 (4.34)	4.36 (4.25)	*p* = 0.197
Depression subscale of DASS-21 (0–42), mean (SD)		5.04 (4.60)	4.71(6.07)	*p* = 0.543

^1^ Normally distributed data—tested with the Student’s *t*-test. All the other continuous variables were not normally distributed and were tested with the Mann–Whitney U test.

**Table 4:** Odds ratios (OR) of possible factors associated with pelvic girdle pain using conditional multiple regression.

	OR (95%CI)	*p*-Value
Vaginal resting pressure	0.702 (0.502–0.981)	**0.0383**
Diastasis recti severity (classified as mild, moderate or severe)	11.060 (0.222–550.555)	0.2280
Correct activation of pelvic floor muscles (yes)	0.104 (0.004–2.637)	0.1698

## Data Availability

The raw data used to support the conclusions of this article are available from the respective corresponding author upon request.

## Appendix A

**Table A1:** STROBE Checklist.

	Item No.	Recommendation	Page No.
**Title and abstract**	1	(*a*) Indicate the study’s design with a commonly used term in the title or the abstract	1
		(*b*) Provide in the abstract an informative and balanced summary of what was done and what was found	1
**Introduction**			
Background/rationale	2	Explain the scientific background and rationale for the investigation being reported	1–2
Objectives	3	State specific objectives, including any prespecified hypotheses	2
**Methods**			
Study design	4	Present key elements of study design early in the paper	2
Setting	5	Describe the setting, locations, and relevant dates, including periods of recruitment, exposure, follow-up, and data collection	3
Participants	6	(*a*) Give the eligibility criteria, and the sources and methods of case ascertainment and control selection. Give the rationale for the choice of cases and controls	3
		(*b*) For matched studies, give matching criteria and the number of controls per case	2
Variables	7	Clearly define all outcomes, exposures, predictors, potential confounders, and effect modifiers. Give diagnostic criteria, if applicable	3–5
Data sources/measurement	8 *	For each variable of interest, give sources of data and details of methods of assessment (measurement). Describe comparability of assessment methods if there is more than one group	3–5
Bias	9	Describe any efforts to address potential sources of bias	4–5
Study size	10	Explain how the study size was arrived at	5
Quantitative variables	11	Explain how quantitative variables were handled in the analyses. If applicable, describe which groupings were chosen and why	5
Statistical methods	12	(*a*) Describe all statistical methods, including those used to control for confounding	5
		(*b*) Describe any methods used to examine subgroups and interactions	n/a
		(*c*) Explain how missing data were addressed	5
		(*d*) If applicable, explain how matching of cases and controls was addressed	5
		(*e*) Describe any sensitivity analyses	n/a
**Results**			
Participants	13 *	(a) Report numbers of individuals at each stage of study—e.g., numbers potentially eligible, examined for eligibility, confirmed eligible, included in the study, completing follow-up, and analysed	5
		(b) Give reasons for non-participation at each stage	3
		(c) Consider use of a flow diagram	n/a
Descriptive data	14 *	(a) Give characteristics of study participants (e.g., demographic, clinical, social) and information on exposures and potential confounders	Table 1 Table 2
		(b) Indicate number of participants with missing data for each variable of interest	5
Outcome data	15 *	Report numbers in each exposure category, or summary measures of exposure	Table 3
Main results	16	(*a*) Give unadjusted estimates and, if applicable, confounder-adjusted estimates and their precision (e.g., 95% confidence interval). Make clear which confounders were adjusted for and why they were included	Table 4
		(*b*) Report category boundaries when continuous variables were categorized	Table 3
		(*c*) If relevant, consider translating estimates of relative risk into absolute risk for a meaningful time period	n/a
Other analyses	17	Report other analyses done—e.g., analyses of subgroups and interactions, and sensitivity analyses	n/a
**Discussion**			
Key results	18	Summarise key results with reference to study objectives	8
Limitations	19	Discuss limitations of the study, taking into account sources of potential bias or imprecision. Discuss both direction and magnitude of any potential bias	11–12
Interpretation	20	Give a cautious overall interpretation of results considering objectives, limitations, multiplicity of analyses, results from similar studies, and other relevant evidence	8–11
Generalisability	21	Discuss the generalisability (external validity) of the study results	12
**Other information**			
Funding	22	Give the source of funding and the role of the funders for the present study and, if applicable, for the original study on which the present article is based	12

* Give information separately for cases and controls.
